# Cost-consequence analysis of continuous denosumab therapy for osteoporosis treatment in South Korea

**DOI:** 10.1186/s12891-024-07185-8

**Published:** 2024-01-20

**Authors:** Seungju Cha, Minjeong Sohn, Hyowon Yang, Eric J. Yeh, Ki-Hyun Baek, Jeonghoon Ha, Hyemin Ku

**Affiliations:** 1 NDnex, Saebitgongwon-ro 67, Gwangmyeong-si, Gyeonggi-do 14348 Republic of Korea; 2 Amgen Korea Ltd, Seoul, Republic of Korea; 3 Amgen Inc, Thousand Oaks, CA USA; 4 Internal Medicine, Yeouido St.Mary’s Hospital, College of Medicine, The Catholic University of Korea, Seoul, Republic of Korea; 5 Internal Medicine, Seoul St.Mary’s Hospital, College of Medicine, The Catholic University of Korea, Seoul, Republic of Korea

**Keywords:** Osteoporosis, Denosumab, Osteoporotic fracture, Bone density, Cost-consequence analysis, Republic of Korea

## Abstract

**Background:**

Insurance reimbursement provisions in South Korea limit osteoporosis medication availability for patients with T-scores exceeding − 2.5. This study aimed to evaluate the financial impact and fracture prevention of continuous denosumab therapy until a T-score>-2.0 (Dmab-C strategy), versus discontinuation of denosumab after reaching T-score>-2.5 (Dmab-D strategy) in osteoporosis patients.

**Methods:**

A cost-consequence analysis from a Korean healthcare system perspective was performed using a newly developed Markov model. The incidence of vertebral and non-vertebral fracture, fracture-related deaths, drug costs, and fracture-treatment costs were estimated and compared between Dmab-C and Dmab-D strategy over a lifetime in eligible patients aged 55 years.

**Results:**

Base-case analysis revealed that Dmab-C prevented 32.21 vertebral fracture (VF) and 12.43 non-VF events per 100 patients over a lifetime, while reducing 1.29 fracture-related deaths. Lifetime direct healthcare cost saving per patient was KRW 1,354,655 if Dmab-C replaces Dmab-D. When productivity losses were considered, Dmab-C saved KRW 29,025,949 per patient compared to Dmab-D. The additional treatment costs of Dmab-C could be offset by the higher subsequent treatment costs and fracture treatment costs of Dmab-D. The sensitivity analysis showed consistent patterns with results of the base-case analysis.

**Conclusion:**

Continuous treatment using denosumab until osteoporosis patients achieve and maintain a T-score of -2.0 would provide greater clinical and economic benefits in terms of fracture prevention and reduced mortality risks compared to outcomes from discontinuing treatment at a T-score of -2.5 or above. This new treatment strategy would effectively lower the risk of fractures and fracture-related mortality, ultimately leading to lower medical expenses.

**Supplementary Information:**

The online version contains supplementary material available at 10.1186/s12891-024-07185-8.

## Background

Osteoporosis is a systemic skeletal disease, that causes reduced bone mass, which makes the bones fragile [1]. Osteoporosis primarily causes fragility fractures associated with low trauma in the elderly [2]. Osteoporotic fractures are associated with unfavorable health outcomes, such as decreased mobility [3], extended hospital stays, worse quality of life [4], increased mortality [5], and a significant economic burden. Osteoporosis patients in South Korea increased from 0.86 million in 2016 to 1.05 million in 2020 and are expected to increase every year. It is estimated that over 50% of the prevalence of osteoporosis in Korea is in 60 + age groups, and due to the aging society, the incidence of osteoporotic fractures in patients over 50 years old has been rapidly increasing from 186,488 since 2008 to 275,131 in 2016 [6, 7]. According to the Korean National Health and Nutrition Examination Survey, the total number of osteoporotic fractures and associated medical expenses increased by 28.9% and 31.6%, respectively, from 2008 to 2011 [8]. From an insurance perspective, the total cost of osteoporotic fractures in Korea was estimated at $722 million in 2011 [8]. The economic burden of osteoporotic fractures is expected to rise with the increase in the life expectancy and elderly population in South Korea [9]. Consequently, owing to the increased financial burden in the healthcare systems, osteoporosis and associated fractures have become a major concern for all stakeholders, especially payers.

Denosumab, a fully human monoclonal antibody, is the first approved biologic agent for the treatment of osteoporosis. The nuclear factor kappa B ligand (RANKL) receptor can be targeted to decrease bone resorption and boost bone mineral density (BMD). The phase 3 FREEDOM and FREEDOM Extension studies, which were randomized controlled trials investigating the relationship between the BMD T-score and fracture outcomes in postmenopausal women treated with denosumab for over 3 years and 10 years respectively, have demonstrated the efficacy of denosumab in increasing BMD and decreasing fracture rates [10–12]. The AACE/ACE guideline for the treatment of osteoporosis recommends denosumab as an initial therapy, which should be continued if clinically necessary without a drug holiday [13].

The Korea Food and Drug Administration authorized denosumab for the treatment of osteoporosis in 2014, which has been reimbursed as a first-line treatment since 2019. Patients who have a BMD T-score of less than − 2.5 are eligible for Korea national insurance benefits twice a year while patients with osteoporotic fractures are eligible for insurance benefits six times for 3 years. BMD monitoring is recommended once a year to follow-up the treatment effectiveness, and extended reimbursement therapy beyond the insurance coverage duration is restricted to patients with BMD T-score remaining below − 2.5 according to the current Korea reimbursement guidelines. Unfortunately, osteoporosis patients who has recovered their T-scores just above the − 2.5 (e.g., between − 2.0 and − 2.5) are no longer covered by insurance and therefore likely to discontinue denosumab. In such case, the patient’s bone mass may not have increased sufficiently to prevent osteoporotic fractures, and the subsequent decline in the BMD T-score after treatment discontinuation may increase the risk of osteoporotic fractures including risks of multiple fractures. Unlike the Korean reimbursement guideline, international guidelines, such as AACE/ACE guidelines, recommended the persistence of osteoporosis diagnosis even after subsequent DXA shows T-score higher than − 2.5, if the initial osteoporosis diagnosis is made according to T-score of -2.5 or below [13]. The guideline also recommended continuing treatment without holiday for non-bisphosphonate antiresorptive drugs, including denosumab, if clinically appropriate, and strongly recommend that patients with osteoporosis should continue taking the medications even after achieving T-score over − 2.5 [13]. These restrictions would likely lead to a discrepancy in treatment strategies between international guidelines, such as the AACE/ACE guideline recommendations, versus clinical practice observed in the real-world setting in Korea as Korea is one of the few countries which limit the use of osteoporotic drugs using T-score, while other countries do not limit the treatment duration. We hypothesized that continuous reimbursement coverage of denosumab until T-score of -2.0 in osteoporosis patients who recovered to -2.5 < T-score≤-2.0 would lead to favorable cost-consequence profiles.

Therefore, this study aimed to evaluate the long-term cost-consequence of Dmab-C (denosumab-continuation) strategy, which is continuous denosumab treatment for osteoporosis patients with T-score improved from below − 2.5 to -2.5 < T-score≤-2.0, compared to the current strategy where patients discontinued denosumab and all osteoporotic drugs after reaching a T-score of -2.5 (Dmab-D; denosumab-discontinuation), in the study population of adults aged 55 years from the Korean healthcare system perspective. Originally, continuous treatment should be guaranteed without a BMD T-score limit, however, considering the government’s financial burden, our study analyzed the clinical and cost benefits when treatment was extended to at least T-score − 2.0.

## Methods

### Model overview

**Fig. 1:**
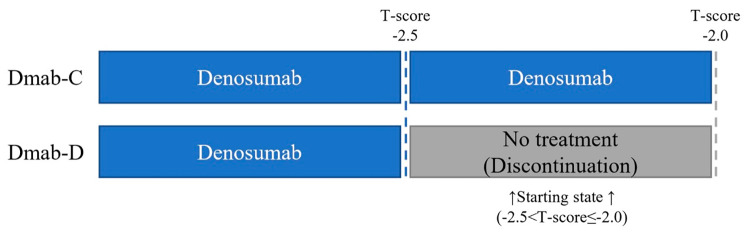
Study population

We conducted a cost-consequence analysis (CCA), a type of economic evaluation method assessing the costs and outcomes for each of the alternatives separately [14]. CCA evaluates a wide range of costs, including direct costs (e.g., medications, hospitalizations), indirect costs (e.g., lost productivity, caregiving cost), and intangible costs (e.g., pain, suffering), alongside multiple outcomes such as clinical effects, patient-reported impacts, and societal implications. Unlike other cost-effectiveness evaluation methods that merges data into a single metric (QALYs, ICER etc.), CCA uniquely presents costs and consequences separately, allowing decision-makers to thoroughly analyze each aspect independently [15]. The predefined study population were patients with osteoporosis aged 55 years whose BMD T-score had improved from below − 2.5 to -2.5 < T-score≤-2.0 after denosumab treatment (Fig. 1).

BMD T-score in this analysis was based on the T-score measured at the total hip. The Dmab-C strategy is defined when denosumab treatment continued until T-score reached − 2.0. The Dmab-D strategy is defined when denosumab and all osteoporotic drugs were discontinued once the T-score reached − 2.5.

Based on a review of the literature, clinical guidelines, and clinical expert opinion [10, 12, 16, 17], a Markov model was developed to reflect the natural history of the disease in the target population with considerations of changes in T-scores and incidence of fractures. Markov model offers a dynamic framework to simulate the progression of health states and associated costs and outcomes over time. In CCA, the Markov model segments a population into discrete health states and transitions individuals between these states based on predefined probabilities. By capturing the complexities of disease progression and treatment effects over time, the Markov model in CCA enables the estimation of long-term costs and outcomes associated with different interventions [18].

Key study outcomes/consequence included total costs per patient, number of vertebral fractures (VF), number of non-vertebral fractures (non-VF), and fracture-related deaths per 100 patients over a lifetime horizon. The Korean healthcare system perspective and a 4.5% discount rate were used for both cost and consequences, as recommended by the Korean Guidelines for Pharmacoeconomic Evaluation [19]. The model cycle was defined as 6 months in line with the denosumab treatment cycle, and all the input values were calculated according to 1 cycle.

### Model structure

**Fig. 2:**
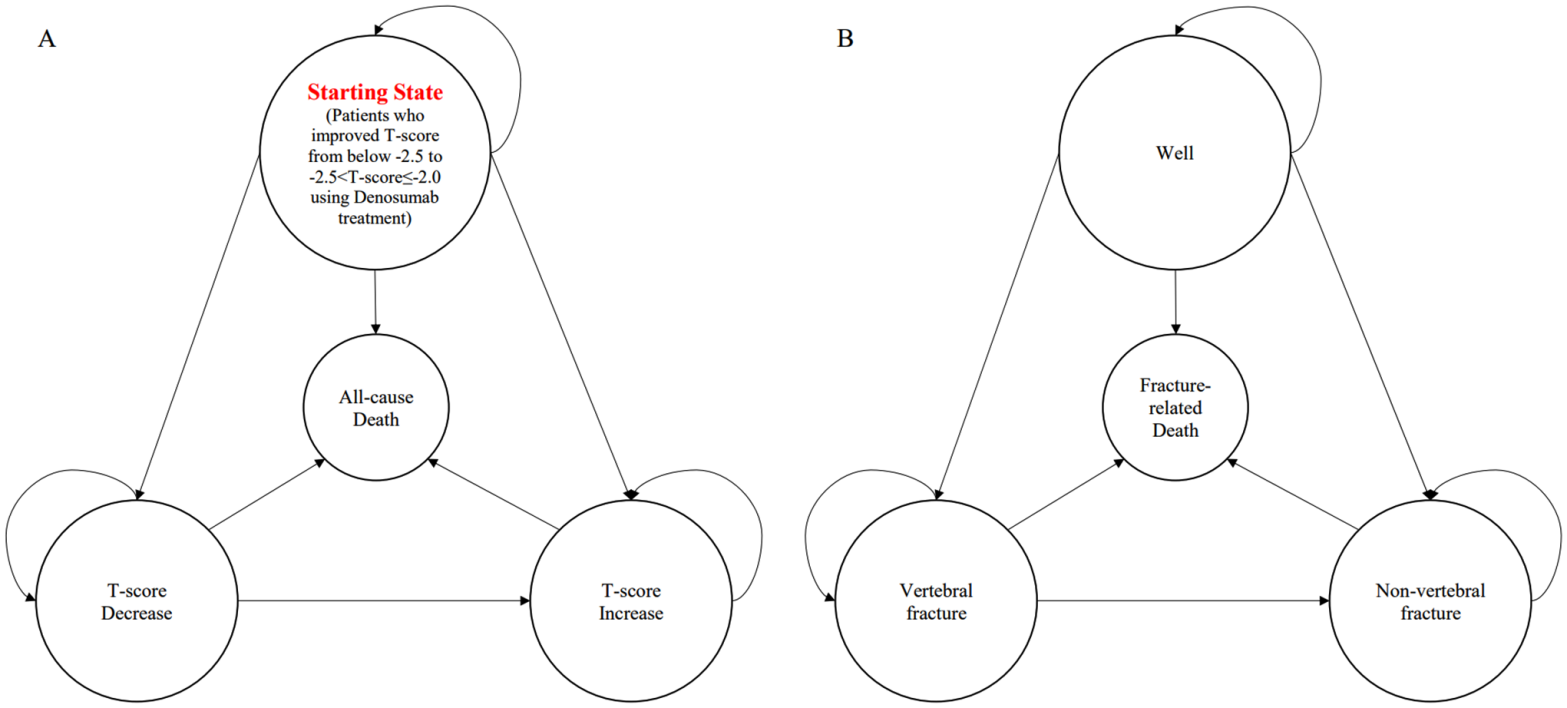
Model structures. **A**. Main health state. (1) Starting state: Patients whose BMD T-score improved from below − 2.5 to -2.5 ≤ T-score<-2.0 with denosumab; (2) T-score decrease: Patients who rebound to BMD T-score below − 2.5; (3) T-score increase: Patients who T-score increased over − 2.0 or over − 2.5 ≤ T-score<-2.0 after subsequent therapy; (4) All-cause death; **B**. Sub-health state. Sub-health state probabilities differed according to the main health state. (1) Well (no new fracture); (2) Vertebral fracture; (3) Non-vertebral fracture; (4) Fracture-related death

A Markov model was generated using Microsoft Excel (Microsoft 365 (Office), Microsoft corp.) to simulate and analyze the transitions and outcomes within the model. The Markov model is comprised of four main health states that reflect the patient’s BMD T-score status (Fig. 2A) and four sub-health states reflecting fracture (Fig. 2B). The main health states included the Starting state (state S), the T-score decrease state (state D), the T-score increase state (state I), and all-cause death (death). All patients initiated the Markov model in the state S, where their BMD T-scores had increased to between − 2.5 and − 2.0 after receiving denosumab treatment, representing an improvement due to treatment from the initial T-score below − 2.5. The state D was defined as patients who had a decrease of T-Score below − 2.5 from the state S. Patients in state D state were assumed to receive subsequent treatment, using denosumab or bisphosphonate (BP) or selective estrogen receptor modulator (SERM). The state I was stratified into two categories: (1) patients whose BMD T-score increased over − 2.0 from state S or from state D after receiving subsequent treatment and (2) patients whose BMD T-score increased over − 2.5 after receiving subsequent treatment from state D. We assumed that once patients transitioned into the T-score increase state, no further health state transition occurs except death.

The model also incorporated sub-health states that represented the fracture incidence within each main health states. These sub-health states were classified into four states: well (no fracture), vertebral fracture, non-vertebral fracture, and fracture-related death.

### Probabilities and key assumptions

The transition probabilities (TP) between health states considering T-score were obtained primarily from the FREEDOM and FREEDOM Extension studies [10, 12]. The TP between the states of the Dmab-C strategy was calculated by analyzing the proportion of patients who attained T-score of >-2.5 and >-2.0 after 3 years from FREEDOM baseline [12]. The TP from state S to state D of the Dmab-C strategy was presumed to differ between on-treatment and off-treatment. We presumed that in the case of the Dmab-C strategy, the duration of denosumab treatment is 3 years, considering the denosumab treatment duration and persistence confirmed through real-world data. During the 3 years of denosumab treatment, we assumed that patients do not transit from state S to state D [20–22]. However, after the 3 years of denosumab treatment, we assumed that the treatment effect gradually decreases over 2 years of “offset time”, in which patients’ T-score rebounded to below − 2.5 [23, 24]. The TP from state S to state I was calculated from the percentage of patients who attained T-score>-2.0 from − 2.5 < T-score≤-2.0 at baseline [12]. The TP from the state D to the state I was calculated by a weighted average of the TP of subsequent treatments, including denosumab, BP, and SERM, considering the Korean market share. The TP for denosumab treatment from state D to state I was calculated from the proportion of patients who attained − 2.5 < T-score≤-2.0 from ≤-2.5 at baseline [12]. TP of BP and SERM treatments were estimated by multiplying the RR (Risk ratio) from a prior network meta-analysis study by the TP of the denosumab treatment [25].

The TP from state S to state D in Dmab-D strategy was also calculated by considering the offset time. As the Dmab-D strategy discontinued all osteoporotic drugs, including denosumab, it was assumed that patients no longer had an increase in T-score, meaning the TP from state S to state I is 0. The TP from state D to state I was estimated by multiplying the RR of placebo vs. denosumab from the FREEDOM study by the TP of the Dmab-C strategy [10].

### Incidence of fracture and mortality

In each health state (S, I, D), patients undergo sub-health states including well, vertebral fracture, non-vertebral fracture, and fracture-related death. The fracture probabilities in health state S, I and D of Dmab-C strategy were derived from the FREEDOM and FREEDOM extension studies from the expected 1-year fracture incidence of each T-score range [10, 12]. The fracture probabilities in health state S, I and D of the Dmab-D strategy were estimated by multiplying the RRs by the fracture probabilities of the Dmab-C strategy. We used the RRs reported in the indirect and mixed treatment comparison study for osteoporosis drugs [25]. The TPs of the BMD T-score and fracture rates are listed in Table 1.

**Table 1:** Summary of model settings and input parameters

Variables	Model input		Reference
	Dmab-C	Dmab-D	
**General population characteristics**			
Age(years)	55		Assumption
**General analysis settings**			
Cycle length	6 months		-
Time horizon	Lifetime		-
Discount rate	4.5%		[17]
Market share in patients with T-score decrease (Denosumab)	50.9%		IQVIA(2021, Q3)
Market share in patients with T-score decrease (BP/SERM)	49.1%		IQVIA(2021, Q3)
**T-score Transition Probability (per cycle)**			
From Starting state^a^ to T-score decrease (0–3 years)^b^	0.000	0.822	[12, 21]
From Starting state^a^ to T-score decrease (after 3 years)^c^	0.822	0.822	[12]
From Starting state^a^ to T-score increase	0.108	0.000	[12]
From T-score decrease to T-score increase	0.033	0.033	[12]
**Fracture probability** ^d^ **(per cycle)**			
Starting state^a^	0.004	0.013	[10, 12]
T-score decrease	0.006	0.019	
T-score increase (-2.5 < T-score≤-2)	0.004	0.013	
T-score increase (-2.0 < T-score)	0.002	0.007	
**Non-vertebral fracture**			
Starting state^a^	0.013	0.016	[10, 12]
T-score decrease	0.015	0.018	
T-score increase (-2.5 < T-score≤-2)	0.013	0.016	
T-score increase (-2.0 < T-score)	0.009	0.011	
**Costs (per cycle, KRW)**			
**Drug costs**			
Denosumab	177,650		HIRA, Weighted average drug price list, 2021
BP(Oral)	125,112		
BP(IV)	107,296		
SERM	120,050		
**Administration costs**			
Denosumab	4,843		HIRA, Fee-for-service price, 2022
BP(Oral)	20,226		
BP(IV)	2,422		
SERM	45,705		
**Fracture treatment costs**			
Vertebral fracture	2,717,095		[26]
Non-vertebral fracture	2,684,098		

Dmab-C: continuous denosumab therapy, Dmab-D: discontinuation of denosumab; ^a^Patients whose T-score recovered to -2.5 < T-score≤-2.0 from ≤-2.5 using denosumab treatment; ^b^Transition probability during continuous treatment with denosumab in Dmab-C; ^c^Transition probability after continuous treatment (3 years); TP after 3 years of continuous denosumab treatment duration; ^d^*Dmab − D fracture probability = Dmab − C fracture proability × RRs*

Mortality differs by the sub-health state of the patients. General mortality information was obtained from the Korean life table provided by the Korean Statistical Information Service (KOSIS). General mortality was applied to patients who are in a ‘well’ sub-health state, which means the patients have no fracture. The standard mortality ratio (SMR) was applied to patients who are in the sub-health state of ‘vertebral fracture’ or ‘non-vertebral fracture’, based on the study by Lee et al. for VF and Yoo et al. for non-VF [26, 27]. The SMR used in the model is listed in Table 2.

**Table 2:** Standardized mortality ratio (SMR)

Fracture status		SMR	Reference
Mortality of the general population		Reference	KOSIS
No new fracture		1	-
Vertebral fracture^a^	Male	4.56	[24]
	Female	2.99	
Non-vertebral fracture^b^	Male	5.02	[25]
	Female	3.29	

^a^SMR at 6 months after vertebral fracture in men and women; ^b^Yoo et al. presented the mortality HR (hazard ratio) of non-VF compared to that of VF [humerus (1.41), wrist (0.52), and hip (1.68)]. The final non-VF SMR was calculated by multiplying the SMR od VF by the weighted HR of non-VF compared to VF; VF: vertebral fracture; KOSIS: Korean Statistical Information Service

### Costs

The direct medical costs considered in the model included drug costs, administration costs, and fracture treatment costs. Drug and administration costs were extracted from the reimbursement price list and fee-for-service price list of the Health Insurance Review & Assessment Service (HIRA) of South Korea using micro-costing methods. Fracture treatment costs were estimated using the VF and non-VF (hip, wrist, and others) costs reported in the 2017 Osteoporosis and Osteoporotic Fracture Fact Sheet provided by the Korean Society for Bone and Mineral Research and National Health Insurance Service (NHIS) joint research [16, 28]. All costs used to inform model inputs were estimated and adjusted accounting for inflation to the year 2022 in Korean Won (KRW).

### Analysis

For each strategy, the cumulative fractures (i.e., VF and non-VF) and fracture-related deaths per 100 patients were estimated, and medical costs (i.e., continuous drug costs, subsequent drug costs, fracture treatment costs) per person were calculated over a lifetime horizon. The disaggregated results of costs and outcomes are presented in both discounted and undiscounted forms to ensure straightforward comparison between Dmab-C and Dmab-D. One-way sensitivity analyses (OWSA) were conducted to evaluate the impact of assumptions and uncertainty of input values on base-case analysis conditions. OWSA considered input values with various levels of uncertainty for age, time horizon, denosumab treatment duration, offset time after denosumab discontinuation, fracture costs, and inclusion of costs due to productivity losses. OWSA was performed by individually varying one factor at a time. Productivity loss was calculated based on the human capital approach by multiplying age-specific average income and employment rates with the productivity life years lost by patients due to osteoporotic fractures [29].

Additional sensitivity analysis was conducted to compare Dmab-C strategy with continuous oral BP treatment (BP-C (Oral)) strategy, continuous IV BP treatment (BP-C (IV)) strategy and continuous SERM treatment (SERM-C) strategy for osteoporosis patients whose T-score recovered from below − 2.5 to -2.5 < T-score≤-2.0 with denosumab treatment [25, 30–33]. The duration of BP-C and SERM-C treatments was also limited to 3 years as was the base-case analysis. The model inputs for the BP-C and SERM-C analysis are presented in Supplementary Table S1.

## Results

### Base-case analysis

Table 3 shows key results from the base-case analysis. Compared to the Dmab-D strategy, the Dmab-C strategy would reduce the total number fractures by 46.64 fractures per 100 patients (VF 34.21; non-VF 12.43), the number of fracture-related deaths by 1.29 per 100 patients. The total lifetime medical costs in patients on Dmab-C strategy were KRW 1,354,655 lower than those in patients on Dmab-D. Despite the additional cost of denosumab treatment in the Dmab-C (KRW 717,120), KRW 808,651 would be saved as a result of preventing additional subsequent treatments in state D (T-score below − 2.5), and additional KRW 1,263,124 would be saved as reduced fracture treatment costs. (Table 3).

**Table 3:** Key results from the base-case analysis (Discounted, undiscounted)

Variables	Discounted			Undiscounted		
	Dmab-C	Dmab-D	Difference	Dmab-C	Dmab-D	Difference
Total fracture^a^	54.01	100.65	-46.64	98.55	179.88	-81.33
Vertebral fracture	13.89	48.09	-34.21	25.01	84.49	-59.48
Non-vertebral fracture	40.13	52.56	-12.43	73.54	95.38	-21.85
Fracture-related death^a^	1.76	3.06	-1.29	5.77	9.82	-4.04
Total lifetime costs^b^	4,017,571	5,372,225	-1,354,655	6,432,703	8,796,976	-2,364,273
Continuous drug cost^b^	717,120	0	717,120	754,004	0	754,004
Subsequent drug cost^b^	1,846,137	2,654,788	-808,651	3,025,230	3,940,990	-915,760
Fracture treatment cost^b^	1,454,314	2,717,437	-1,263,124	2,653,470	4,855,986	-2,202,517

Dmab-C: continuous denosumab therapy; Dmab-D: denosumab discontinuation (discontinuation of denosumab when the T-score improved from below − 2.5 to -2.5 < T-score≤-2.0 after denosumab treatment); ^a^per lifetime in 100 patients; ^b^per patient, KRW

### Sensitivity analysis

Table 4 presents key results of OWSA. The findings of OWSA demonstrate the robustness of the analysis. We observed that fracture incidence and cost were consistently lower in Dmab-C strategy compared to Dmab-D strategy across all OWSA scenarios. Notably, the top 2 sensitive factors were the time horizon and productivity loss. When a societal perspective is considered, the inclusion of the productivity loss would lead to additional cost saving of KRW 27,663,741 in comparison with costs from the payer’s perspective.

Additional sensitivity analysis comparing Dmab-C strategy with either the BP-C (Oral) or BP-C (IV) strategy or SERM-C strategy presented similar patterns (Supplementary Table S2). Compared to the BP-C (Oral) strategy, the Dmab-C strategy would reduce the total number of 6.19 fractures (VF 3.07; non-VF 3.12), 0.14 in fracture-related deaths per 100 patients, and KRW 607,061 as the total lifetime medical costs per patient. Compared to the BP-C (IV) strategy, total number of 7.14 fractures (VF 1.90; non-VF 5.24), 0.15 in fracture-related deaths per 100 patients, and KRW 466,391 was reduced per patient during lifetime. Similarly, compared to the SERM-C strategy, the Dmab-C strategy would reduce the number of 22.76 fractures (VF 16.81; non-VF 5.94), 0.619 in fracture-related deaths per 100 patients, and KRW 747,194 as the total lifetime medical costs per patient.

**Table 4:** Key results of one-way sensitivity analysis

Analysis		Difference (Dmab-C - Dmab-D)								
		Fracture risks			Mortality	Costs				
		VT^a^	Non-VT^a^	Total ^a^	Fracture-related deaths^a^	Continuous treatment drug^b^	Subsequent treatment drug^b^	Total fracture treatment^b^	Productivity loss^b^	Total lifetime^b^
Base-case		-34.21	-12.43	-46.64	-1.29	717,120	-808,651	-1,263,124	-	-1,354,655
Discount rate	0%	-59.48	-21.85	-81	-4.04	754,004	-915,760	-2,202,517	-	-2,364,273
	3%	-40.23	-14.67	-55	-1.83	728,943	-839,036	-1,486,710	-	-1,596,804
Time horizon	3 years	-6.68	-2.48	-9.16	-0.06	717,120	-667,236	-247,984	-	-198,100
	5 years	-10.80	-3.87	-14.67	-0.10	717,120	-718,177	-397,194	-	-398,251
	10 years	-18.77	-6.65	-25.42	-0.21	717,120	-762,596	-688,520	-	-733,996
Starting age	72.3^c^	-23.94	-8.37	-32.31	-2.56	705,908	-755,078	-875,038	-	-924,208
Offset time	1 year	-34.23	-12.43	-46.66	-1.29	717,120	-812,642	-1,263,741	-	-1,359,263
	3 years	-34.18	-12.43	-47	-1.29	717,120	-803,556	-1,262,323	-	-1,348,758
Continuous treatment duration^d^	1 year	-32.82	-10.28	-43	-1.20	165,561	-181,090	-1,167,781	-	-1,183,310
	2 years	-33.57	-11.44	-45	-1.25	460,590	-516,956	-1,219,014	-	-1,275,380
	5 years	-35.24	-14.02	-49	-1.36	1,133,895	-1,281,661	-1,333,693	-	-1,481,460
	Lifetime	-38.05	-18.18	-56	-1.67	2,364,007	-2,654,788	-1,521,662	-	-1,812,443
Continuous treatment until T-score − 1.5^e^	-2.5 < T-score≤-1.5	-34.58	-12.74	-47.32	-1.33	745,271	-683,387	-1,281,513	-	-1,219,629
Perspective	Societal	-34.21	-12.43	-46.64	-1.29	717,120	-808,651	-1,263,124	-27,663,741	-29,025,949

Dmab-C: continuous denosumab therapy; Dmab-D: denosumab discontinuation (discontinuation of denosumab when the T-score improved from below − 2.5 to -2.5 < T-score≤-2.0 after denosumab treatment); VF: vertebral fracture; non-VF: non-vertebral fracture; ^a^per lifetime in 100 patients; ^b^KRW, per patient lifetime; ^c^Average age in the FREEDOM trial; ^d^Duration of continuous denosumab treatment in the Dmab-C group

## Discussion

To the best of our knowledge, this is the first study to analyze and compare the costs and consequences between the two strategies of continuous denosumab treatment and discontinuation of denosumab in patients whose T-score recovered from less than − 2.5 to -2.5 < T-score≤-2.0 after denosumab treatment. Our study indicates that the strategy of continuous denosumab treatment would potentially reduce the incidence of VF and non-VF, reduce fracture-related mortality, and lead to significant cost savings primarily driven by reduced fracture treatment costs.

The model was developed to represent the treatment pathways for the osteoporosis population in Korea. The model is similar to the previously published cost-effectiveness models in terms of considering fractures as a main health status to compare the different treatment strategies [24, 34, 35]; The key difference is that the current model incorporated the T-score to the main health state and was designed to compare the cost-consequence between the strategies due to the fractures resulted by the T-score change, while other models were designed to compare the cost-effectiveness between drugs regardless of T-score change.

Expert opinions and previous studies suggest that 20–40% of osteoporosis patients experience a fracture during their lifetime [36], and those with a fracture history have an increased risk of subsequent fractures over 5-years period, up to 30% [37]. In the present study, the expected lifetime fracture incidence per 100 patients were 54.01 and 100.65 for Dmab-C and Dmab-D, respectively. As our study considered multiple fractures at different sites and subsequent fractures in patients with a fracture history as separate event, it can be evaluated that the expected lifetime fractures from our study were reasonably estimated.

In the current study, the denosumab discontinuation strategy would lead to increased incidence of VF and non-VF during the lifetime, which may contribute to an additional 1.29 fracture-related deaths per 100 patients. Our findings are consistent with conclusion from previous studies, i.e., discontinuation of not only denosumab, but also the majority of osteoporotic medications, such as BP, SERM, led to an increase in the fracture risk, despite improvements in the T-scores [38–40].

The cost of denosumab treatment would likely be offset by lifetime cost savings primarily due to the reduced fracture treatment costs as a result of reduced incidence of fractures. Our study estimated that, compared to the Dmab-D strategy, the Dmab-C strategy would reduce costs by KRW 1,354,655, which is equivalent to approximately 4.93% of the additional per capita healthcare cost (i.e., KRW 27,492,295) in patients with one or more fractures at age 55 compared with the general population with no fracture in Korea [29]. These findings suggest that the Dmab-C strategy would lead to significant cost savings. Previous Pharmacoeconomics studies have shown that persistent use of osteoporotic medications, including denosumab, can decrease fracture-related medical costs [35, 41, 42].

The primary objective of the study was to compare Dmab-D with Dmab-C. However, in real-world scenarios, patients may receive BP or SERM as alternative treatments to maintain T-score after surpassing − 2.5. While the BP-C and SERM-C strategies showed diminished effectiveness in reducing fractures and cost savings compared to Dmab-C (Supplementary Table S2), they demonstrated fracture reduction and cost-efficiency when compared to patients who discontinued treatment(Dmab-D). BP-C and SERM-C strategies would reduce 23.8 to 28.9 total number of fractures, 0.58 to 0.79 fracture-related deaths per 100 patients and save KRW 434,156 to 607,460 as the total lifetime medical expense per patient. This suggests that overall osteoporosis treatments (Denosumab, BP, SERM) could merit consideration upon Korea’s insurance extension to T-score≤-2.0.

Osteoporotic fractures impose a substantial burden on productivity, leading to significant losses across various domains, including absenteeism, presenteeism, and caregiving. The consequences of these fractures extend beyond the immediate physical toll, often resulting in prolonged periods of impaired functionality and decreased work capacity. This hindrance to productivity is not limited solely to the affected individuals but also affects their caregivers, workplaces, and the broader socioeconomic landscape. The cumulative effect of these fractures on productivity underscores the critical importance of preventive measures and proactive management strategies to alleviate the substantial societal and economic impact caused by osteoporosis-related fractures [9, 43, 44]. With the potential implementation of the continuous denosumab treatment strategy, a reduction in medical expenses of KRW 709.8 billion would be expected, based on an assumption of 524,018 osteoporosis patients receiving the Dmab-C treatment strategy in South Korea in 2022.

There are several limitations and key assumptions in our study. First, the current model assumed that the risk ratio of patients on Dmab-D was similar to that in the placebo group of the FREEDOM study. This assumption was made due to insufficient BMD data and is subject to a limitation. South Korea is one of the few countries that limits reimbursement coverage of osteoporotic medications based on BMD T-score. Such restrictions likely have an impact on the availability of real-world evidence comparing fracture incidences between patients with BMD values of -2.5 < T-score≤-2.0 who continue and discontinue denosumab treatment. In addition, our Markov health states were based on the T-score instead of BMD level (g/cm^2^) or BMD percentage changes. While clinical references predominantly rely on BMD for fracture probability calculations, we utilized T-score-based fracture risk models to derive estimates for our study. Consequently, this approach inevitably introduced limitations in our ability to estimate fracture probabilities. Second, our model assumed that T-score rebound can occur only once from the starting state, and that the health state remains static once patients reach T-score increase. However, it is still possible for patients to experience multiple rebounds to T-score≤-2.5. This could be another limitation. Third, the model could not capture the history of fractures, which may be an important factor affecting recurrent fracture probabilities in patients with osteoporosis.

Despite the above-mentioned limitations, this is the first study to provide evidence to support that continuous denosumab treatment is clinically and economically beneficial for osteoporosis patients whose T-score recovered from below − 2.5 to -2.5 < T-score≤-2.0 with denosumab treatment. The current healthcare system in Korea limits insurance coverage of denosumab for osteoporosis and allows denosumab coverage only for patients whose T-score remains below − 2.5 after 1 year or 3 years of treatment. However, the primary goal of osteoporosis treatment is to prevent osteoporotic fractures, which requires patients to continuously receive proper osteoporosis medications for an extended period of time despite T-score reaching above − 2.5 in order to facilitate a substantial increase in bone mass. Otherwise, the benefits of increased bone mass from previous denosumab treatment may be lost after denosumab being discontinued. Based on the findings in our study, we would recommend the strategy of continuous administration of denosumab should be considered in clinical practice in South Korea; this strategy also consistent with the recommendations from AACE/ACE guidelines.

This study also demonstrated the potential lifetime cost-savings and reduction in total fracture and fracture-related deaths as a result of the proposed strategy with continuous denosumab treatment compared to the current strategy with reimbursement restrictions. Other countries, including the U.S., Australia, and Japan, have no restrictions on the treatment reimbursement period of anti-osteoporotic drugs. Importantly, our study results indicate potential lifetime cost savings, so lifting the current restriction of limiting denosumab treatment to osteoporosis patients only with T-score≤-2.5 would likely have a positive financial forecast for the Korean health systems. The crucial evidence from our study could be used by public health policymakers and healthcare providers to make informed treatment decisions to allow persistent treatment of osteoporosis when T-score beyond − 2.5 in order to reduce the risk of sequential osteoporotic fractures and attendant economic burden.

## Conclusion

This study indicates that the strategy of continuous denosumab treatment in osteoporosis patients whose T-score improved from below − 2.5 to -2.5 < T-score≤-2.0 would reduce medical costs and fracture incidence. Therefore, evidence-based continuous treatment using denosumab until osteoporosis patients achieve and maintain a T-score − 2.0 can be considered in clinical practice in South Korea. Further studies on health-related quality of life and cost-effectiveness analysis will be helpful to promote informed decision-making.

### Electronic supplementary material

Below is the link to the electronic supplementary material.

Supplementary Material 1

## Data Availability

The data mainly used in this cost-consequence study are available from published data, which are referenced next to each input used in this study. General mortality in Koreans is available from KOSIS (Korean Statistical Information Service; https://kosis.kr/index/index.do). Data sharing is no applicable to this article as no datasets were generated or analyzed during the current study.

## Abbreviations

BMD: Bone Mineral Density
DXA: Dual-Energy X-ray Absorptiometry
AACE/ACE: American Association of Clinical Endocrinologists and American college of Endocrinology
Dmab-C: Denosumab-continuation
Dmab-D: Denosumab-discontinuation
CCA: Cost-consequence Analysis
VF: Vertebral Fracture
Non-VF: non-vertebral Fracture
TP: Transition Probability
BP: Bisphosphonate
SERM: Selective Estrogen Receptor Modulator
SMR: Standard Mortality Ratio
OWSA: One-way Sensitivity Analysis
