# Supplementary material for: Cost-consequence analysis of continuous denosumab therapy for osteoporosis treatment in South Korea
Source: BMC Musculoskelet Disord. 2024 Jan 20;25:76. doi: 10.1186/s12891-024-07185-8 (PMC10799461; doi:10.1186/s12891-024-07185-8)
Supplement: Supplementary file 1 — Supplementary Material 1 [file 12891_2024_7185_MOESM1_ESM.docx]

| **Supplementary Table S1. Transition and Fracture probability Input in the BP-CR, SERM-CR models** | | | | |
| --- | --- | --- | --- | --- |
| **Variables** | **Model input** | | | **Reference** |
|  | **BP-C (Oral)** | **BP-C (IV)** | **SERM-C** |  |
| **T-score Transition Probability (per cycle)** | | | | |
| Starting state^a^ → T-score decrease (0-3 years)^b^ | 0.005 | 0.005 | 0.822 | [12, 21] |
| Starting state^a^ → T-score decrease (after 3 years)^c^ | 0.822 | 0.822 | 0.822 | [12] |
| Starting state^a^ → T-score increase | 0.000 | 0.000 | 0.000 | [12] |
| T-score decrease → T-score increase | 0.033 | 0.033 | 0.033 | [12] |
| **Fracture probability (per cycle)** | | | | |
| **Vertebral fracture** | | | | |
| Starting state^a^ | 0.007 | 0.005 | 0.008 | [10,12, 23] |
| T-score decrease | 0.006 | 0.006 | 0.012 |  |
| T-score increase(-2.5<T-score≤-2) | 0.004 | 0.004 | 0.008 |  |
| T-score increase(-2.0<T-score) | 0.002 | 0.002 | 0.005 |  |
| **Non-vertebral fracture** | | | | |
| Starting state^a^ | 0.014 | 0.018 | 0.014 | [10, 12, 23] |
| T-score decrease | 0.015 | 0.015 | 0.016 |  |
| T-score increase(-2.5<T-score≤-2) | 0.013 | 0.013 | 0.014 |  |
| T-score increase(-2.0<T-score) | 0.009 | 0.009 | 0.009 |  |
| ^a^Patients whose T-score recovered to -2.5<T-score≤-2.0 from T-score≤-2.5 with denosumab treatment; ^b^TP during continuous treatment using BP or SERM in the starting state; ^c^TP after discontinuation of continuous treatment (after first 3 years): BP-C, SERM-C off treatment; BP-C: continuous bisphosphonate therapy, SERM-C: continuous selective estrogen receptor modulator therapy | | | | |

| **Supplementary Table S2. Sensitivity analyses Results (Discounted results)** | | | | | | | |
| --- | --- | --- | --- | --- | --- | --- | --- |
| **Variables** | **Discounted** | | | | **Difference (Dmab-C - Comparator)** | | |
|  | **Dmab-C** | **BP-C (Oral)** | **BP-C (IV)** | **SERM-C** | **BP-C (Oral)** | **BP-C (IV)** | **SERM-C** |
| Total fracture^a^ | 54.01 | 60.20 | 61.15 | 76.77 | -6.19 | -7.14 | -22.76 |
| Vertebral fracture | 13.89 | 16.95 | 15.79 | 30.70 | -3.07 | -1.90 | -16.81 |
| Non-vertebral fracture | 40.13 | 43.25 | 45.36 | 46.07 | -3.12 | -5.24 | -5.94 |
| Fracture-related death^a^ | 1.76 | 1.90 | 1.91 | 2.38 | -0.14 | -0.15 | -0.619 |
| Total lifetime costs^b^ | 4,017,571 | 4,626,961 | 4,486,291 | 4,764,765 | -609,390 | -468,720 | -747,194 |
| Continuous treatment drug cost^b^ | 717,120 | 675,327 | 509,803 | 35,494 | 41,793 | 207,317 | 681,626 |
| Subsequent treatment drug cost^b^ | 1,846,137 | 2,330,163 | 2,330,013 | 2,658,565 | -484,026 | -483,876 | -812,428 |
| Total fracture treatment cost^b^ | 1,454,314 | 1,621,471 | 1,646,474 | 2,070,706 | -167,157 | -192,161 | -616,392 |
| Dmab-C: continuous denosumab therapy; BP-C: continuous BP therapy (BP treatment when the T-score improved from below -2.5 to -2.5<T-score≤-2.0 after denosumab treatment); SERM-C: continuous SERM treatment (SERM treatment when the T-score improved from below -2.5 to -2.5<T-score≤-2.0 after denosumab treatment); ^a^per lifetime in 100 patients; ^b^per patient, KRW | | | | | | | |

| **Supplementary Table S3. Sensitivity analyses Results (Undiscounted results)** | | | | | | | |
| --- | --- | --- | --- | --- | --- | --- | --- |
| **Variables** | **Undiscounted** | | | | **Difference (Dmab-C - Comparator)** | | |
|  | **Dmab-C** | **BP-C (Oral)** | **BP-C (IV)** | **SERM-C** | **BP-C (Oral)** | **BP-C (IV)** | **SERM-C** |
| Total fracture^a^ | 98.55 | 108.25 | 109.25 | 137.83 | -9.70 | -10.69 | -39.28 |
| Vertebral fracture | 25.01 | 29.32 | 28.09 | 54.04 | -4.31 | -3.07 | -29.03 |
| Non-vertebral fracture | 73.54 | 78.93 | 81.16 | 83.79 | -5.39 | -7.62 | -10.25 |
| Fracture-related death^a^ | 5.77 | 6.18 | 6.18 | 7.71 | -0.40 | -0.41 | -1.94 |
| Total lifetime costs^b^ | 6,432,703 | 7,438,100 | 7,289,848 | 7,703,967 | -1,005,397 | -857,145 | -1,271,264 |
| Continuous treatment drug cost^b^ | 754,004 | 711,342 | 536,990 | 35,775 | 42,662 | 217,013 | 718,228 |
| Subsequent treatment drug cost^b^ | 3,025,230 | 3,811,580 | 3,811,335 | 3,950,872 | -786,350 | -786,105 | -,925,642 |
| Total fracture treatment cost^b^ | 2,653,470 | 2,915,179 | 2,941,523 | 3,717,320 | -261,709 | -288,053 | -1,063,850 |
| Dmab-C: continuous denosumab therapy; BP-C: continuous BP therapy (BP treatment when the T-score improved from below -2.5 to -2.5<T-score≤-2.0 after denosumab treatment); SERM-C: continuous SERM treatment (SERM treatment when the T-score improved from below -2.5 to -2.5<T-score≤-2.0 after denosumab treatment); ^a^per lifetime in 100 patients; ^b^per patient, KRW | | | | | | | |
